# Electron beam-based fabrication of crosslinked hydrophilic carbon electrodes and their application for capacitive deionization†

**DOI:** 10.1039/c8ra10527h

**Published:** 2019-03-26

**Authors:** Hyo-Sub Kim, Joon-Yong Sohn, In-Tae Hwang, Junhwa Shin, Chan-Hee Jung, Won Keun Son, Kyung Suk Kang

**Affiliations:** Research Division for Industry and Environment, Korea Atomic Energy Research Institute 29 Geumgu-gil Jeongeup-si Jeollabuk-do 56212 Republic of Korea jch@kaeri.re.kr +82 63 570 3090 +82 63 570 3064; Department of Energy Engineering, Hanyang University 222 Wangsimni-ro, Seongdong-gu Seoul 04763 Republic of Korea; Siontech 167-2 Techno 2-ro, Yuseong-gu Daejeon 34025 Republic of Korea

## Abstract

In this research, we demonstrated that a crosslinked hydrophilic carbon electrode with better electrochemical performance than hydrophobic counterparts can easily be produced using room-temperature, quick electron-beam irradiation with a hydrophilic methacryloyl-substituted polyvinyl alcohol (SPVA) binder. The SPVA binder was effectively synthesized by trans-esterification of PVA with glycidyl methacrylate. The hydrophilic carbon electrode cast on a graphite sheet from a slurry of activated carbon (AC) and SPVA was irradiated with an electron beam to form a crosslinked structure. The analytical results in terms of the morphology, solvent resistance, chemical composition, and contact angle revealed that the carbon electrode was completely crosslinked by electron-beam irradiation even at the dose of 100 kGy (irradiation time = 180 s). The new electrode exhibited superior water-wettability due to the hydrophilic functionality of SPVA. Furthermore, the hydrophilic carbon electrode with an AC : SPVA composition of 90 : 10 and an absorbed dose of 200 kGy, exhibited a specific capacitance of 127 F g^−1^ (67% higher than the hydrophobic poly(vinylidene fluoride) (PVDF)-based counterpart with the same composition). The specific capacitance was further improved to 160 F g^−1^ with an increase in the AC content. The hydrophilic carbon electrode exhibited noticeably better desalination efficiency than the hydrophobic PVDF-based counterpart.

## Abstract 2

A crosslinked hydrophilic carbon electrode with better desalination performance can easily be produced using room-temperature, quick electron-beam irradiation.

## Graphical abstract

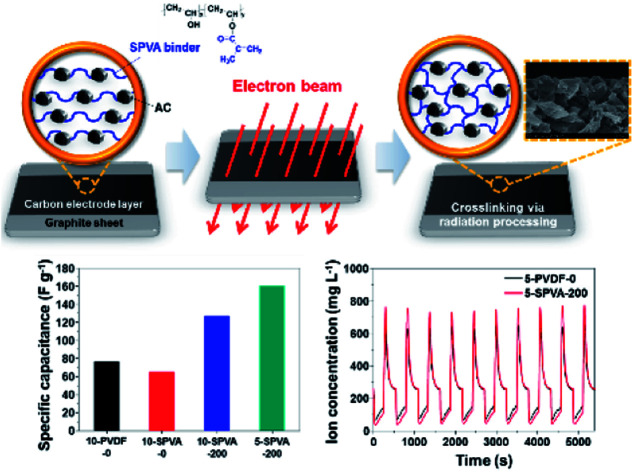

## 1. Introduction

Capacitive deionization (CDI) is an emerging, cost-effective desalination technology that has attracted enormous attention in recent decades.^1–3^ This technology can remove dissolved salts from brackish water using reversible electro-adsorption within an electrical double layer (EDL) formed at the interface between a solution and the electrodes.^4,5^ Among the main components of CDI, the electrodes are regarded as critical because their specific capacitance and stability affect the performance and lifetime of the device.^6^

CDI electrodes have been fabricated using a wide variety of carbon materials including activated carbon, carbon fiber, carbon nanotubes, and carbon black. This is because these carbon materials possess outstanding electrochemical stability, high electrical conductivity, large surface area, and good wettability.^7–13^ To achieve a carbon electrode, polymer binder is necessary to bind the carbon materials. Typically, hydrophobic polymers including poly(vinylidene fluoride) (PVDF) and poly(tetrafluoroethylene) (PTFE) are used as binders in the fabrication of the electrodes due to their high dielectric property, good binding capability, and electrochemical stability.^14–19^ However, their hydrophobicity reduces the wettability of the electrode and hinders ions moving into the pores. This results in decreased ion adsorption capacity of the electrodes, which is critically associated with the capacitive deionization performance.^20^

In this respect, hydrophilic polymers have been considered promising alternatives. When carbon electrodes are fabricated using hydrophilic polymer, chemical crosslinking is a necessary procedure because the hydrophilic polymer is dissolved in water.^21,22^ Moreover, this crosslinking method commonly requires crosslinking agents, long-processing time, and high temperatures. Therefore, there is still high demand for a cross-linking method that occurs at room temperature, and is also quick, cost-effective, and scalable.

The electron beam technique is a powerful approach for the crosslinking of hydrophilic carbon electrodes.^23^ This radiation processing offers several clear advantages over conventional crosslinking processes, including operation at room temperature, solid-state chemical reaction without additives (initiator), higher throughput rates, and more precise control over the process.^23^ For these reasons, this technique has been widely used in the manufacturing of various industrial products including tire cord, heat-resistant electrical cable, polymer fuse, and polymer foam.^24^ Despite these benefits, there has been no report on preparation of crosslinked hydrophilic carbon electrodes by combination of the electron beam technique with radiation-crosslinkable hydrophilic binder, for use in the CDI process.

In this work, we intended to develop a crosslinked hydrophilic carbon electrode by electron beam irradiation of a methacryloyl-substituted polyvinyl alcohol (SPVA) binder and to demonstrate its applicability toward ion exchange membrane-based CDI. The radiation-crosslinkable hydrophilic SPVA was synthesized using a trans-esterification of PVA with glycidyl methacrylate (GMA). To elucidate the formation of the crosslinked hydrophilic carbon electrodes, SPVA-based carbon electrodes of different compositions were irradiated with electron beams to achieve various absorbed doses. In systematic comparison with the conventional hydrophobic PVDF-based ones with the same composition, the newly-developed SPVA-based carbon electrodes at the absorbed dose of 200 kGy exhibited a gel fraction of 100% for better solvent resistance, an immeasurable water contact angle for better water wettability, and a 67% higher specific capacitance. As a result, the SPVA-based electrode exhibited better desalination performance (desalination efficiency, salt adsorption capacity and charge efficiency) than conventional PVDF-based ones. Therefore, these findings demonstrate that radiation crosslinking with a hydrophilic polymer binder could provide a simple, cost-effective, and scalable method for the fabrication of high-performance carbon electrodes for waste water treatment and energy storage.

## 2. Experimental

### 2.1. Materials

Poly(vinyl alcohol) (PVA, 99+% hydrolyzed powder, *M*_w_ = 130 000), poly(vinylidene fluoride) (PVDF, *M*_w_ = 530 000), and glycidyl methacrylate (GMA, 97%) were obtained from Sigma-Aldrich (USA). Dimethyl sulfoxide (DMSO, 99%) and *N*,*N*-dimethylacetamide (DMAc, 99%) were purchased from Showa Chemicals Inc. (Japan). Commercial MSP20 activated carbon and graphite sheet as a collector were purchased from Kansai Cokes and Chemicals (Japan) and Dongbang Carbon Corp. (Korea), respectively. All the chemicals were used as received.

### 2.2. Synthesis of SPVA binder

As shown in the scheme of synthesis (Fig. S1†), the SPVA was prepared by trans-esterification of PVA with GMA in a polar solvent according to the literature, where this reaction was carried out without any catalyst.^25,26^ Briefly, PVA (8 g) was completely dissolved in DMSO (200 mL) at 80 °C. After naturally cooling down to ambient temperature, a certain molar amount of GMA with respect to the OH functionalities of PVA was added to the homogenous PVA solution (where the molar ratios of GMA : PVA were 0.10, 0.15, 0.18, and 0.20), and then stirred gently for 6 h at 60 °C. After the trans-esterification reaction was complete, the mixture was cooled to ambient temperature, and then slowly poured into pure, cool acetone to obtain the SPVA product. The resulting product was washed three times with pure acetone and then dried in a vacuum oven at 50 °C for 2 d.

### 2.3. Preparation of cross-linked SPVA-based electrode by electron beam irradiation

Carbon electrodes of different types and compositions were prepared using a slurry casting technique described in Table 1. The slurries were first prepared by mixing MSP20 powder (activated carbon) and PVDF or SPVA (polymer binder) in DMAc and then using a planetary centrifugal mixer (ARE-310, Thinky mixer, Japan) for 10 min at 2000 rpm. The resulting slurries were cast on graphite sheets as collectors using a doctor blade (Elcometer®, Belgium). Afterwards, the resulting electrode was dried in a vacuum oven for 4 h at 50 °C to remove the remaining DMAc. For the electron beam irradiation, the prepared SPVA-based carbon electrodes were put into aluminium pouches and thermally sealed after purging with N_2_ gas. The sealed pouches were irradiated at room temperature with an ELV-8 electron beam accelerator installed at EB-Tech (Daejeon, Korea). The energy and current density of the electron beam was 1.5 MeV and 7.2 mA cm^−2^, respectively. The total absorbed dose and irradiation time were (50, 100, and 200) kGy and (90, 180, and 360) s, respectively. To circumvent thermal effects on the samples during irradiation, a sample stage was used as a cooling plate and kept at 5 °C. The cellulose triacetate dosimetry was carried out following ISO/ASTM 51650, and the uncertainty of the doses given by EB-Tech was less than 5%. The total mass of the prepared electrodes was 1.9 g.

**Table 1:** Compositions and absorbed doses of carbon electrodes

Samples	Binders	Composition (wt%)		Absorbed dose (kGy)
		Activated carbon	Binder	
10-PVDF-0	PVDF	90	10	0
5-PVDF-0	PVDF	95	5	0
10-SPVA-0	SPVA	90	10	0
10-SPVA-50	SPVA	90	10	50
10-SPVA-100	SPVA	90	10	100
10-SPVA-200	SPVA	90	10	200
5-SPVA-200	SPVA	95	5	200

### 2.4. Characterization of SPVA-based carbon electrodes

The chemical structure and degree of substitution of SPVA was analyzed using ^1^H-NMR spectroscopy (NMR-400, JEOL, Japan). Deuterated dimethyl sulfoxide (DMSO-d_6_, Sigma-Aldrich) solutions containing 150 mg L^−1^ of sample were used in the NMR analysis. Attenuated total reflectance Fourier transform infrared spectroscopy (ATR-FTIR) analysis was performed using FT-IR spectroscopy (640-IR, Varian, USA) in the range 4000–500 cm^−1^. The morphology and thickness of the electrodes were examined by SEM (scanning electron microscopy, JSM-7500F, JEOL, Japan).

The gel fraction of the electrode was measured by measuring the weight of insoluble parts after solvent extraction in DMSO for 7 d at 50 °C. The gel fraction (*W*_gel_) was calculated using the eqn (1):

1 *W*_gel_ (%) = (*W*_1_ − *W*_g_)/(*W*_0_ − *W*_g_) × 100,

where *W*_0_ is the weight of the dried electrode before the extraction, *W*_1_ is the weight of the electrode after the extraction, and *W*_g_ is the weight of graphite sheet.

The elemental composition of the electrodes was quantified using an X-ray photoelectron spectroscope (XPS, MultiLab 2000, Thermo Electron Corporation, UK) with a monochromatic Mg-Kα source. The water contact angles of the electrodes were measured using a contact angle analyzer (Phoenix 300, Surface Electro Optics, Korea).

The cyclic voltammetry (CV) measurement was performed in three-electrode mode using a potentiostat (VersaSTAT3, AMETEK Inc., USA). A carbon electrode, Pt square plate, and a saturated Ag/AgCl electrode were used as the working electrode, counter electrode, and reference electrode, respectively. The effective surface area of the working electrode was 1.77 cm^2^. The CV measurement was conducted in the potential range (−0.5 to 0.5 V) (*vs.* Ag/AgCl) at a specific scan rate of 5 mV s^−1^. The specific capacitance (*C*, F g^−1^) of the electrodes was calculated using the eqn (2):2
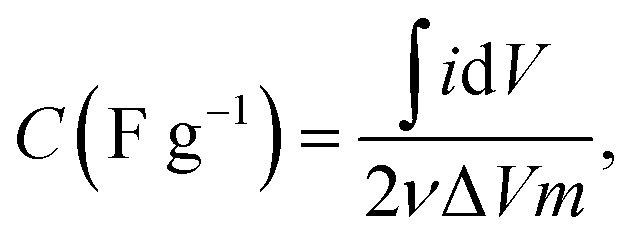
where *i* is the response current (A), *V* is the potential (V), *ν* is the scan rate (V s^−1^) of the test, and *m* is the mass of the electroactive material in the electrode (g). An EIS (electrochemical impedance spectrometry) analysis was conducted using an impedance analyzer (SI-1260, Solartron, UK) in the same three-electrode mode as for the CV measurement. The impedance spectra were obtained in the frequency range 10 mHz to 100 Hz at a potential of 0 V. An alternating sinusoidal signal of 50 mV peak-to-peak was superimposed on the system. Both measurements were carried out in 0.5 M KCl electrolyte.

### 2.5. Desalination performance test

The desalination performance test was performed on a continuous ion exchange membrane-based CDI system consisting of an ion exchange membrane-based CDI unit cell, potentiostat (VersaSTAT4, AMETEK Inc., USA), a peristaltic pump (MASTERFLEX®, Cole-Parmer, USA), and a conductivity meter (CCT-3300, ROC®, China). The unit cell consisted of two parallel porous carbon electrode sheets separated by a non-conductive spacer (nylon cloth, 100 μm thick). The effective surface area of the carbon electrode was 10 cm × 10 cm. A cationic exchange membrane (Neosepta CMX, Astom Co., Japan) and anionic exchange membrane (Neosepta AMX, Astom Co., Japan) were used with the carbon electrode in a CDI device for which 250 mg L^−1^ of NaCl solution was provided to the unit cell at a flow rate of 30 mL min^−1^. Electro-adsorption was performed by applying a potential of 1.2 V for 4 min. After the electro-adsorption experiment, electro-desorption was conducted immediately by applying a potential of −1.2 V for 5 min. The change in the NaCl concentration during the electro-adsorption and desorption process was analysed at the outlet of the unit cell using a conductivity measurement system. On the basis of the linear relationship between NaCl concentration and conductivity, the desalination efficiency (*η*) of the electrode was calculated using the eqn (3):3
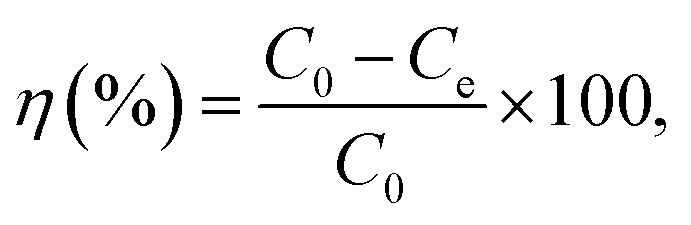
where *C*_0_ is the initial NaCl concentration and *C*_e_ is the effluent concentration during the electro-adsorption and desorption processes.

The salt adsorption capacity (SAC, *Γ*, mg g^−1^) of the electrode was calculated using the eqn (4):4
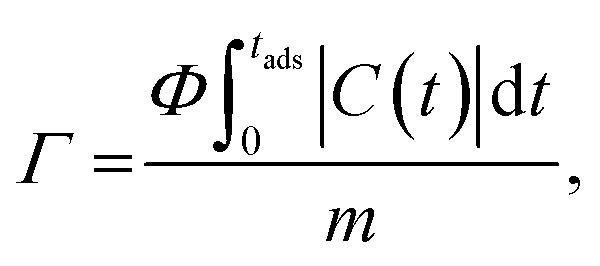
where *Φ* is the volumetric flow rate (L min^−1^), *C*(*t*) is the difference between initial concentration and current concentration (mg L^−1^) as a function of time, *t*_ads_ is adsorption time (min) during the cycle, and *m* is the total mass (g) of electrodes (including activated carbon and binder).

The charge efficiency (*Λ*, %) was calculated using eqn (5):5
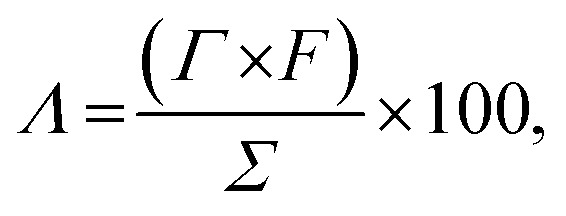
where *Γ* is the SAC, *F* is the Faraday constant (96 485 C mol^−1^), and *Σ* (charge, C g^−1^) is obtained by integrating the corresponding current.

## 3. Results and discussion

For the fabrication of a crosslinked hydrophilic carbon electrode with better electrochemical performance, the SPVA as a radiation-crosslinkable hydrophilic binder enabling rapid, room-temperature crosslinking (unlike with condensation reaction-based crosslinking) was newly synthesized using a well-known trans-esterification reaction with GMA in aprotic solvent, as illustrated in the ESI.† As described in the SPVA characterization details (Fig. S2†), the SPVA with a degree of substitution ranging from 10 to 20 mol% can be produced under the optimized reaction time of 6 h at 60 °C. Unlike the other SPVAs, the one with the substitution degree of 20 mol% was not properly precipitated in the acetone used as a non-solvent to obtain the final product. This indicates that the SPVA substituted at above 20 mol% begins to exhibit the enhanced hydrophobicity responsible for the change in its solubility.^27^ As shown in the gel fraction and swelling degree of the SPVA with the substitution degree of 18 mol% (Fig. S3†), the SPVA exhibited a gel fraction of 100% at the given absorbed dose and its swelling degree gradually declined to 0% with increasing absorbed dose of electron irradiation. Thus, the SPVA with a substitution of 18 mol% was selected as the new radiation-crosslinkable hydrophilic binder for further use in the crosslinked hydrophilic carbon electrodes formed using electron beam irradiation.

### 3.1. Formation of crosslinked hydrophilic SPVA-based carbon electrodes

**Fig. 1:**
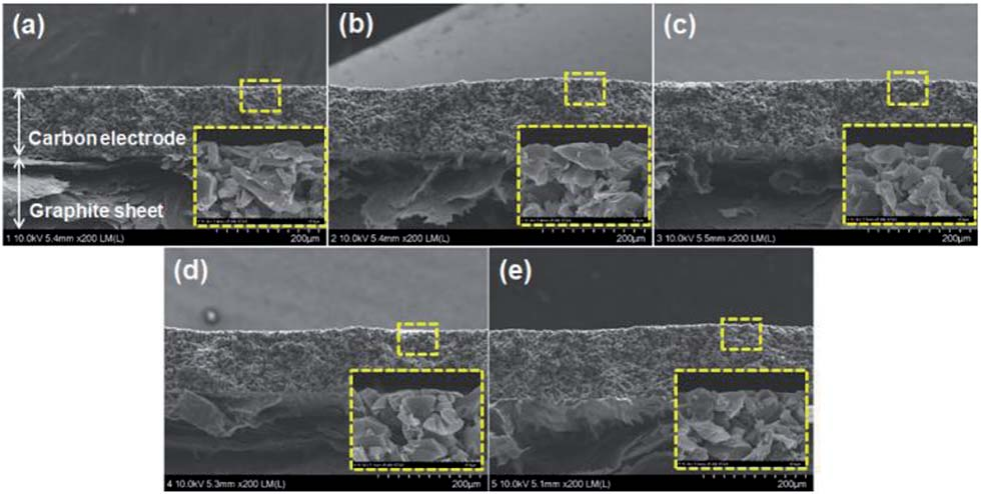
FE-SEM cross-sectional images of carbon electrodes prepared using different binders and absorbed doses: 10-PVDF-0 (a), 10-SPVA-0 (b), 10-SPVA-50 (c), 10-SPVA-100 (d), and 10-SPVA-200 (e). The insets are the magnified images of the dotted squares in each figure.

The changes in the thickness and morphology that occur during the radiation-induced crosslinking of SPVA-based carbon electrodes were investigated by FE-SEM observation. As shown in Fig. 1, all the SPVA-based carbon electrodes (like the PVDF-based one) were uniformly shaped on the graphite collector and their thicknesses was around 135 μm regardless of the absorbed dose. Moreover, as shown in the insets for the magnified images of the SPVA-based carbon electrodes, all the SPVA-based carbon electrodes exhibit the typical morphologies of SPVA binder-mixed activated carbons, and are similar to that of the PVDF-based one. There was no difference in the morphologies of the PVDF and SPVA electrodes, and both PVDF and SPVA binders were well combined with the activated carbon particles. Therefore, this result indicates that no morphological or dimensional transformation occurs in the SPVA-based carbon electrodes during the electron-beam irradiation.

**Fig. 2:**
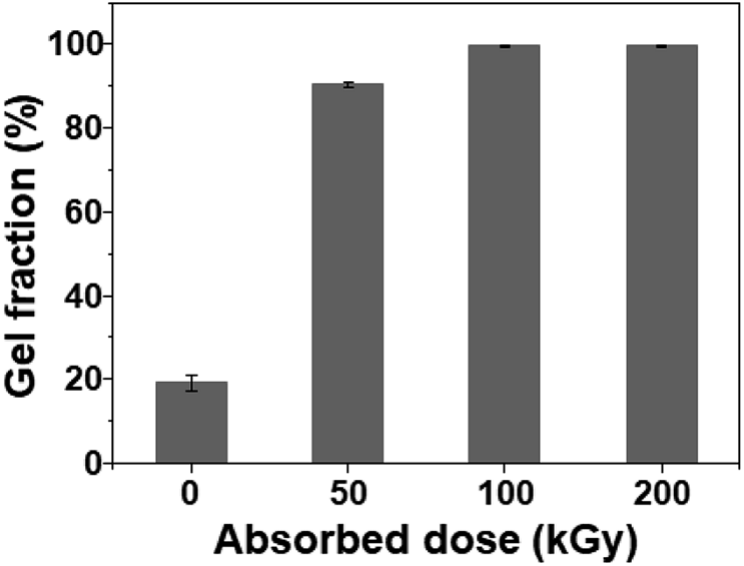
Gel fraction of SPVA-based carbon electrodes with different absorbed doses.

To verify the formation of crosslinked structures in the SPVA-based carbon electrodes during room-temperature electron-beam irradiation, the gel fraction of the SPVA-based carbon electrodes was quantified by measuring the weight of the insoluble part before and after the extraction, using DMSO as a good solvent for the SPVA binder. As shown in Fig. 2, the non-irradiated 10-SPVA-0 exhibited the gel fraction of 19% (originating from a small amount of the remaining graphite collector removed by the solvent extraction). This indicates that the un-crosslinked SPVA binder remaining in the 10-SPVA-0 was definitely dissolved in the given solvent extraction; thereby leading to the 80% disappearance of the carbon electrode on the graphite sheet after the given solvent extraction. On the other hand, the 10-SPVA-100 and 10-SPVA-200 irradiated at room temperature (10-SPVA-50 had 90%) exhibited the gel fraction of 100%, implying that the SPVA binders in the carbon electrodes were completely crosslinked by the electron beam irradiation at absorbed doses above 100 kGy. Moreover, as shown in the photographs of the solvent-extracted carbon electrodes under the same conditions (Fig. S4†), the non-irradiated 10-PVDF-0 and 10-SPVA-0 electrodes (Fig. S4 a and b†). Likewise, the irradiated 10-SPVA-50 exhibited the bright-grey graphite partially removed by solvent extraction (Fig. S4c†). In contrast, as seen in Fig. S4 (d) and (e),† the irradiated 10-SPVA-100 and 10-SPVA-200 manifestly existed as initially formed. Therefore, the electron beam irradiation at absorbed doses above 100 kGy effectively induced the formation of a crosslinked network structure in the SPVA-based carbon electrodes, allowing for outstanding dimensional stability during the solvent extraction at elevated temperature.

**Fig. 3:**
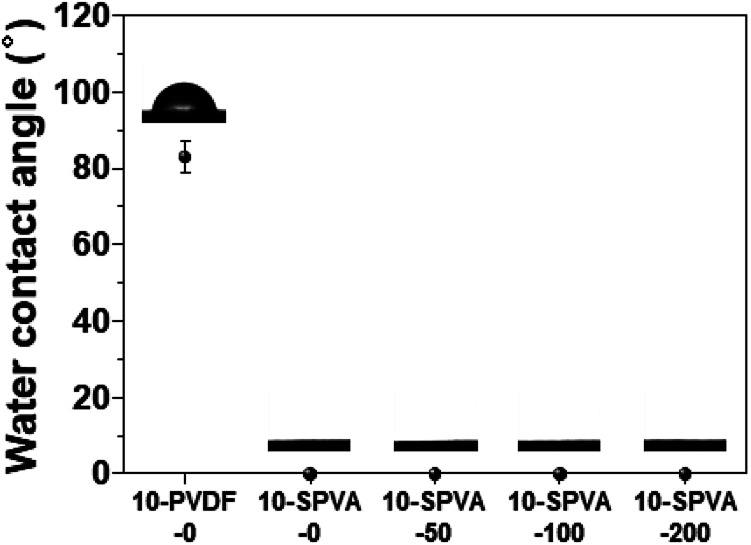
Water contact angles of the carbon electrodes prepared with different binders and absorbed doses: 10-PVDF-0, 10-SPVA-0, 10-SPVA-50, 10-SPVA-100, and 10-SPVA-200.

**Fig. 4:**
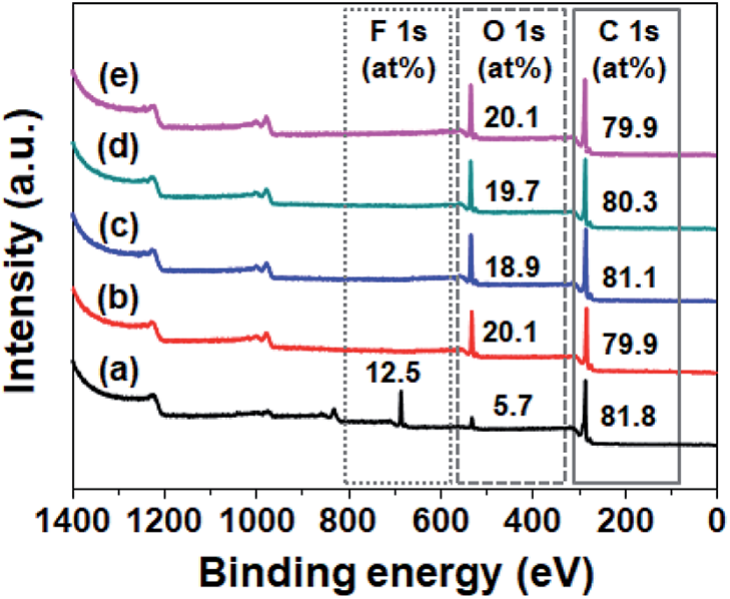
XPS survey spectra of carbon electrodes prepared with different binders and absorbed doses: 10-PVDF-0 (a), 10-SPVA-0 (b), 10-SPVA-50 (c), 10-SPVA-100 (d), and 10-SPVA-200 (e).

To investigate the wettability of the prepared SPVA-based carbon electrodes as one of the important determinants of their electrochemical performance, a static water contact angle measurement was performed. As shown in Fig. 3, the conventional 10-PVDF-0 exhibited the averaged contact angle of 83°, probably due to the presence of the hydrophobic PVDF binder.^28^ On the other hand, all the samples prepared using SPVA binder with the same composition as the PVDF-based carbon electrode (10-SPVA-0, 10-SPVA-50, 10-SPVA-100, and 10-SPVA-200) showed contact angles of 0° regardless of the absorbed dose.^20^ This result indicates that the newly developed SPVA binder (unlike with PVDF) allows the carbon electrodes to be very hydrophilic at the same composition, and that the hydrophilicity of the electrodes is not affected by electron beam irradiation. To provide further clear insight into the surface chemical composition of the SPVA-based carbon electrodes (directly associated with their wettability), XPS was performed. As seen in the XPS survey spectra (Fig. 4(a)), the 10-PVDF-0 spectrum exhibited the typical signals for elemental fluorine (F), oxygen (O), and carbon (C) at 688 eV (12.5 at%), 531 eV (5.7 at%), and 285 eV (81.8 at%), respectively.^29^ The substantial F content, originating only from the PVDF binder in the carbon electrode, seemed to be a key source of the hydrophobicity of the carbon electrodes. In contrast, the non-irradiated 10-SPVA-0 showed the O content of 20.1 at% and C content of 79.9 at%.^30^ This much higher O content (than that in the 10-PVDF-0) stemming from the numerous oxygen-containing functional group of the binders, caused the new carbon electrodes to be more hydrophilic than the PVDF-based one. Moreover, all the PVA irradiated electrodes (10-SPVA-50, 10-SPVA-100, and 10-SPVA-200) had C and O contents similar to those of the non-irradiated one, indicating no significant irradiation-induced changes in chemical composition. Therefore, it turns out from these analytical results that the crosslinked network structure enabled better dimensional stability in aqueous conditions. Moreover, this structure is efficiently induced in the hydrophilic SPVA-based carbon electrodes by quick electron-beam irradiation at room temperature. The resulting carbon electrode exhibits wettability superior to that of the conventional PVDF-based one.

### 3.2. Electrochemical properties of SPVA-based carbon electrodes

**Fig. 5:**
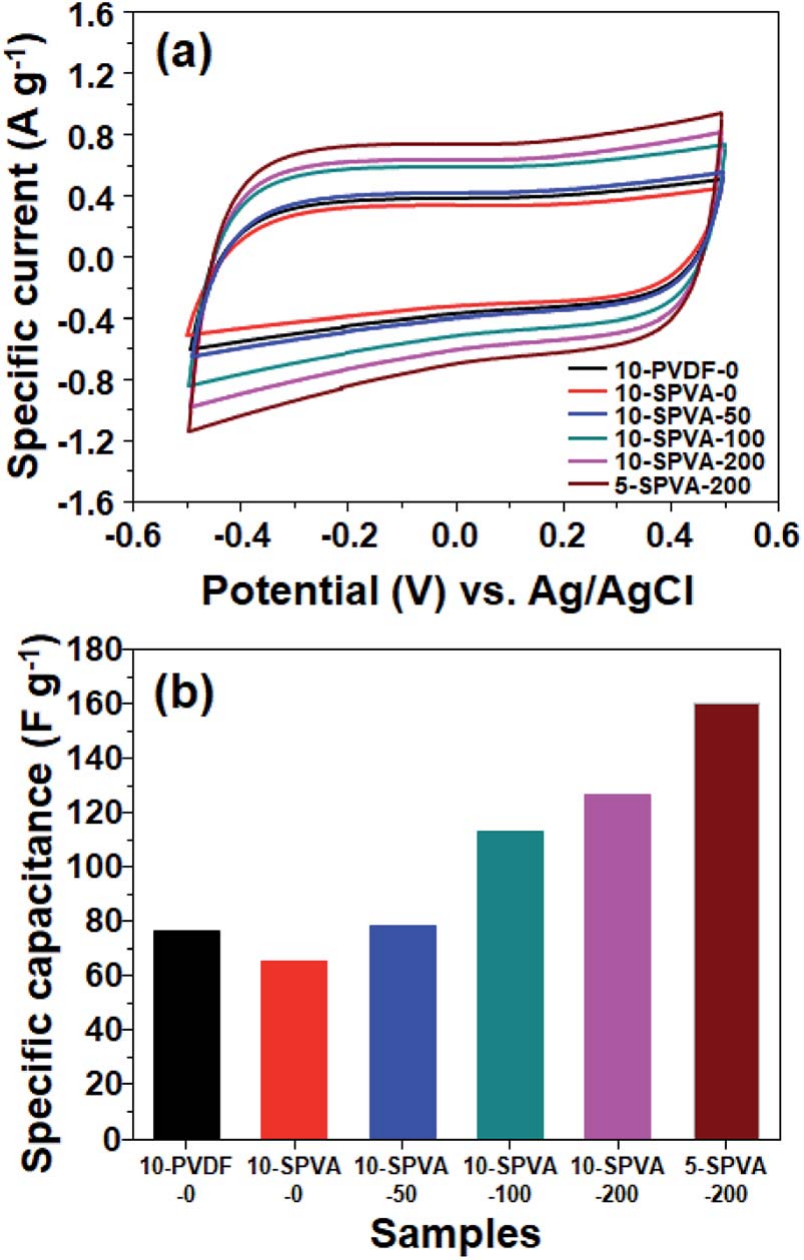
Cyclic voltammograms (a) and their corresponding specific capacitances (b) of carbon electrodes prepared with different binders, composition, and absorbed electron radiation doses in 0.5 M KCl solution at a scan rate of 5 mV s^−1^.

To investigate the effect of hydrophilic SPVA binders and absorbed dose on the specific capacitance of the carbon electrodes, CV charge–discharge analysis was performed using the three-electrode method. As shown in Fig. 5(a), all the prepared SPVA-based carbon electrodes exhibited the typical rectangular-shaped CV curves. These are similar to those of the conventional 10-PVDF-0 within the given range due to the well-known capacitance behavior of the electric double layer on the electrode surfaces, which results are in good agreement with those in previous works.^7,31^ Moreover, as seen in the corresponding specific capacitances of the carbon electrodes calculated from the CV curves (Fig. 5(b)), the non-irradiated 10-SPVA-0 exhibits lower specific capacitance (65 F g^−1^) than that of the conventional 10-PVDF-0 (76 F g^−1^). This is probably due to the swelling phenomenon of the non-crosslinked SPVA binder in the carbon electrodes in the aqueous solution (which reduces the electrical conductivity of the electrodes) as previously reported in the literature.^28,32^ On the other hand, the specific capacitances of the irradiated SPVA-based carbon electrodes increased with increase in the absorbed dose, and 10-SPVA-200 at the higher absorbed dose exhibited higher capacity (127 F g^−1^), corresponding to 67% improvement in comparison to that of 10-PVDF-0. At the same absorbed dose of 200 kGy, the 5-SPVA-200 electrode (containing more of the activated carbon) showed improved specific capacitance of 160 F g^−1^.

This improved specific capacitance for the irradiated SPVA-based carbon electrodes could presumably be explained as follows. Unlike the conventional non-irradiated SPVA-based carbon electrode, the formation of a crosslinking network in the irradiated SPVA-based carbon electrodes allows the hydrophilic SPVA to transport ions efficiently into pores that are blocked in the hydrophobic PVDF-based system, without preventing the SPVA swelling-induced reduction in electrical conductivity, resulting in the improved capacitance.^28,32^ The dose-dependent further improvement is probably due to enhancement of the electrical conductivity caused by the increased crosslinking density of the SPVA binders at the higher dose (leading to the more tightly bound activated carbons).^33,34^ Furthermore, the activated carbon content-dependent improvement result could be ascribed to increment in the surface area and electrical conductivity facilitating the electrochemical processing.^15,16,35^ Therefore, the specific capacitance of the carbon electrode can easily be improved by combining hydrophilic SPVA with quick electron-beam irradiation-induced crosslinking at room temperature, which is beneficial for mass production.

**Fig. 6:**
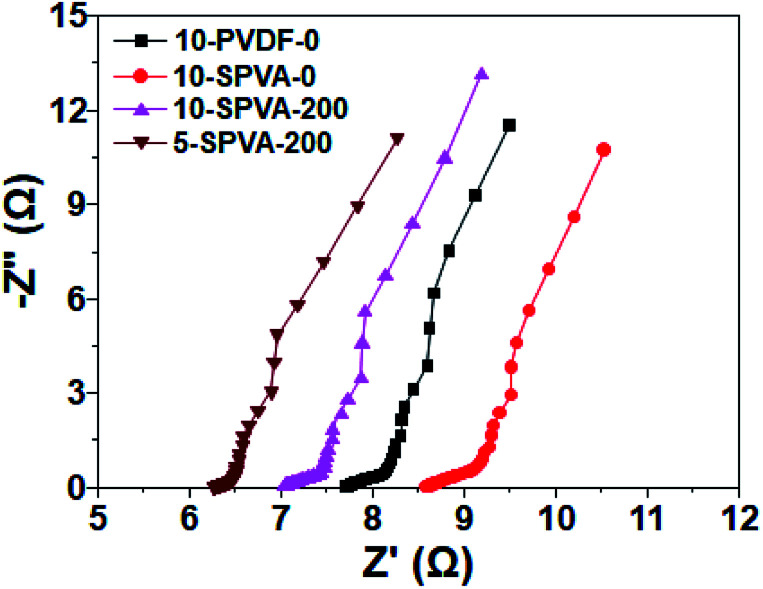
Nyquist plots for the impedance response of 10-PVDF-0, 10-SPVA-0, 10-SPVA-200, and 5-SPVA-200 electrodes.

In conjunction with the CV, EIS analysis was carried out to provide clear insight into the electrochemical behavior of the hydrophilic SPVA-based carbon electrodes prepared by electron beam irradiation. As shown in the EIS Nyquist plots (Fig. 6), the equivalent series resistance of 10-SPVA-0 (8.6 Ω) (taken as the frequency intercept on the real axis corresponding to the contact resistance) is higher than that of the conventional 10-PVDF-0 (7.7 Ω), verifying that reduction in the electrical conductivity of the carbon electrode was caused by the swelling phenomenon of the SPVA binder in aqueous solution, unlike the case with hydrophobic PVDF binder.^28^ On the other hand, unlike the non-irradiated one, the electrical conductivity of the irradiated SPVA-based carbon electrodes had the tendency to increase with increasing absorbed dose. The 10-SPVA-200 electrode exhibited the lowest equivalent series resistance of 7.0 Ω, much lower than even the PVDF-based electrode with the same composition. This indicates increase in the electrical conductivity of the carbon electrode brought about by the irradiation-induced formation of the crosslinking network and its dose-dependent crosslinking density increment.^34^ At the same absorbed dose of 200 kGy, the 5-SPVA-200 (containing higher content of activated carbon) exhibited a lowered equivalent series resistance of 6.3 Ω, providing a reliable clue for the improved electrical conductivity stemming from increase in the amount of electrically conductive activated carbon.^15^ Moreover, the semicircle diameter of the irradiated SPVA carbon electrodes in the high frequency region (corresponding to the interfacial charge transfer resistance) was much reduced, with an increasing absorbed dose and activated carbon content in comparison to those of non-irradiated SPVA- and PVDF-based ones. This supports the notion that the reduced interfacial resistance of the SPVA-based carbon electrodes, crucial to improving the specific capacitance, is also achieved by formation of the crosslinking network, and is further enhanced by increase in the absorbed dose and activated carbon content.^36^ Therefore, the improvement in the electrical conductivity of the SPVA-based electrodes caused by the electron beam irradiation-induced crosslinking, enables the SPVA binders to facilitate better the ion transport into the pores (blocked in the hydrophobic PVDF-based system), giving rise to improvement in the specific capacitance of the carbon electrodes. Among the prepared SPVA-based carbon electrodes, 5-SPVA-200, subjected to the absorbed dose of 200 kGy, exhibited the highest specific capacitance and was used to investigate further the desalination behavior of the new electrode.

### 3.3. Desalination behavior of the SPVA-based carbon electrode

**Fig. 7:**
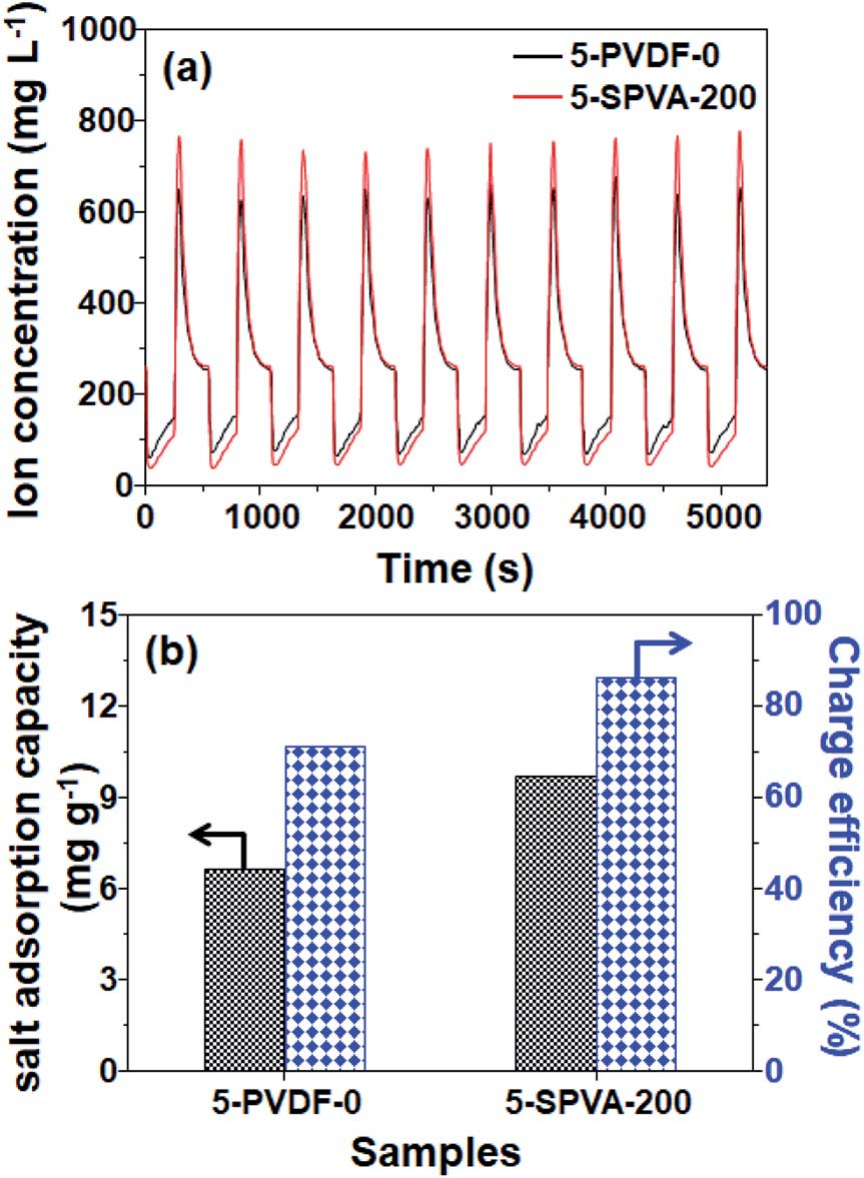
Electrosorption–desorption cycles (a), corresponding salt adsorption capacity (SAC), and charge efficiency (b) of 5-PVDF-0 and 5-SPVA-200 electrodes at the initial NaCl concentration 250 mg L^−1^.

To determine in more detail the effect of the hydrophilic SPVA-based carbon electrodes on salt removal performance, desalination tests were performed with 5-SPVA-200, and with 5-PVDF-0 as a reference for comparison. As shown in Fig. 7(a), both the 5-PVDF-0 and 5-SPVA-200 electrodes exhibited stable cycles of adsorption and desorption over ten cycles, as reported in the literature.^37^ However, more importantly, the 5-SPVA-200 showed better desalination efficiency (68.1%) per adsorption cycle (averaged over ten cycles) than that of the conventional hydrophobic 5-PVDF-0 (51.6%). In addition, the corresponding salt adsorption capacity (indicating the ratio of the mass of adsorbed salt ions to the total mass of electrodes) and charge efficiency (indicating the ratio of the equivalent charge of adsorbed salt ions to the total charge injected into the electrodes during the charging) were evaluated as shown in Fig. 7(b).^3^ The averaged salt adsorption capacity of 5-PVDF-0 and 5-SPVA-200 were 6.63 and 9.69 mg g^−1^, and the averaged charge efficiency of 5-PVDF-0 and 5-SPVA-200 exhibited 71% and 86%. This better desalination performance of the 5-SPVA-200 possibly originates from its two prominent characteristics of higher specific capacitance for ion capture and hydrophilicity for ion uptake, in comparison with that of the hydrophobic PVDF-based carbon electrode.^38^ Therefore, it is firmly believed that the newly developed carbon electrode, obtained by the combination of radiation-crosslinkable hydrophilic SPVA binder with quick electron beam irradiation under ambient conditions, could be considered an efficient electrode for use in the achievement of highly efficient capacitive deionization.

## 4. Conclusions

A crosslinked hydrophilic carbon electrode was prepared using a method that combines quick electron beam irradiation-induced crosslinking under ambient conditions, with the use of hydrophilic SPVA binder. In addition, the new hydrophilic carbon electrode was demonstrated to function well as a desalination electrode. It was clearly confirmed from ^1^H-NMR that the hydrophilic SPVA binder variant with the molar substitution degree of 18 mol% was synthesized by trans-esterification of PVA with GMA. As proved by various analyses in terms of morphology, gel fraction, water contact angle, and chemical composition, the SPVA-based carbon electrode was firmly crosslinked by electron beam irradiation at the absorbed dose of 100 kGy, thereby resulting in the formation of a carbon electrode with outstanding dimensional stability in aqueous solution and excellent hydrophilicity. As a result, the specific capacitance of the irradiated SPVA-based carbon electrode increased to 127 F g^−1^ with increase in the absorbed dose, and could be improved even further to 160 F g^−1^. Importantly, the prepared SPVA-based carbon electrode with the highest specific capacitance exhibited better desalination performances (in terms of desalination efficiency, salt adsorption capacity and charge efficiency) than did the conventional PVDF-based one. The crosslinked hydrophilic carbon electrode was successfully developed and its practical use as a desalination electrode was demonstrated. This study clearly supports the notion that this crosslinked hydrophilic carbon electrode produced by a simple, cost-effective, and scalable method, can be used as a promising carbon electrode applicable for waste water treatment and for energy storage.

## Conflicts of interest

There are no conflicts to declare.

## Supplementary Material

RA-009-C8RA10527H-s001
