# Supplementary material for: Electron beam-based fabrication of crosslinked hydrophilic carbon electrodes and their application for capacitive deionization
Source: RSC Adv. 2019 Mar 26;9(17):9684–91. doi: 10.1039/c8ra10527h (PMC9062162; doi:10.1039/c8ra10527h)
Supplement: RA-009-C8RA10527H-s001 [file RA-009-C8RA10527H-s001.pdf]

Electronic Supplementary Material (ESI) for RSC Advances.
This journal is © The Royal Society of Chemistry 2019

Supporting information

Electron beam-based fabrication of crosslinked hydrophilic carbon electrodes and its application to the capacitive deionization

Hyo-Sub Kim[a], Joon-Yong Sohn[a,b], In-Tae Hwang[a], Junhwa Shin[a], Chan-Hee Jung[a,*], Won Keun Son[c], Kyung Suk Kang[c]

[a] Research Division for Industry and Environment, Korea Atomic Energy Research Institute, 29 Geumgu-gil, Jeongeup-si, Jeollabuk-do 56212, Republic of Korea

[b] Department of Energy Engineering, Hanyang University, 222 Wangsimni-ro, Seongdong-gu, Seoul 04763, Republic of Korea

[c] Siontech, 167-2 Techno 2-ro, Yuseong-gu, Daejeon 34025, Republic of Korea

1. Synthesis of methacryloyl-substituted polyvinyl alcohol (SPVA) binder through trans-esterification with GMA

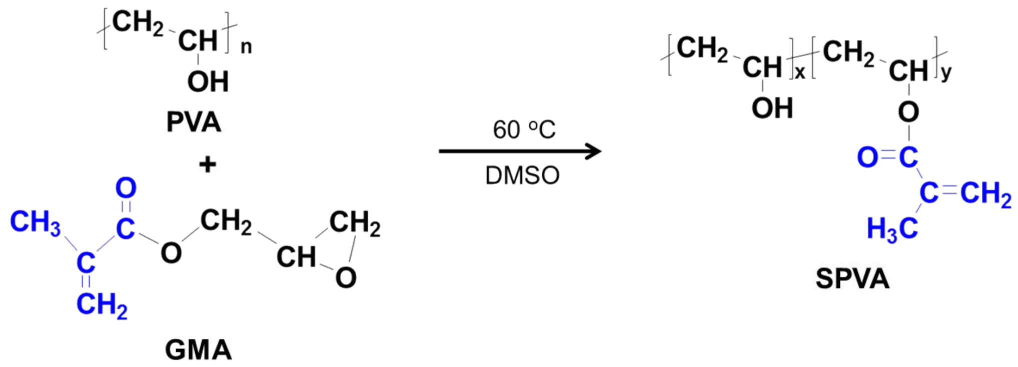

Fig. S1. Scheme for synthesis of SPVA.

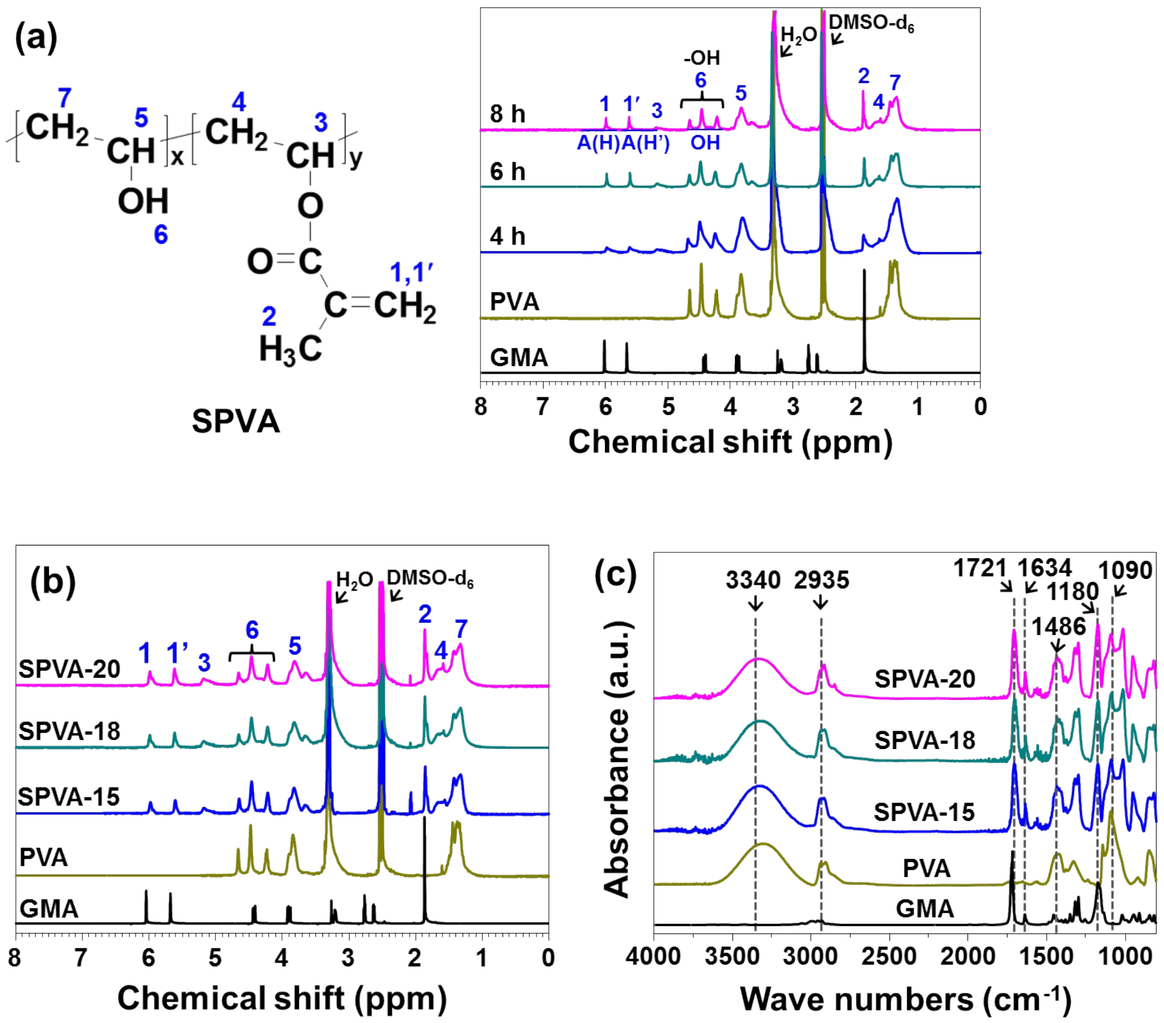

Fig. S2. (a) Molecular structure of SPVA and ^{1}H-NMR spectra of PVA, GMA, and SPVA prepared with different reaction time under the fixed GMA:PVA molar ratio of 0.10, (b) ^{1}H-NMR spectra, and (c) FT-IR spectra of PVA, GMA, and SPVA prepared with different GMA:PVA molar ratios under the fixed reaction time of 6 h (X in SPVA-X means degree of theoretical substitution in mol%).

Table S1. Substitution degree and yield of SPVA with different trans-esterification conditions

GMA:PVA molar ratio	0.10	0.10	0.10	0.15	0.18	0.20
Reaction time (h)	4	6	8	6	6	6
Degree of theoretical substitution (DS_1)[1] (mol%)	10	10	10	15	18	20
Degree of experimental substitution (DS_2)[2] (mol%)	7.9	9.5	9.9	14.8	17.4	19.4
Yield[3] (%)	79	95	99	99	97	97

[1] DS_1 is expected from the reaction molar ratio of GMA:PVA, [2] DS_2 is determined by the ^{1}H-NMR analysis based on ^{1}H-NMR spectrum using the ratio of the averaged area for the vinyl hydrogen from methacryloyl groups at δ5.6 and δ6.0 ppm, and [3] Yield = $DS_2/DS_1 \times 100$.

To optimize the trans-esterification reaction for the preparation of SPVA, the SPVA was synthesized under the different reaction times and GMA:PVA molar ratios, and was characterized by ^{1}H-NMR to quantify the degree of substitution. As shown in the ^{1}H-NMR spectra for the SPVA prepared with different reaction times (Fig. S2 (b) and Table S1), the new peaks (absent in the PVA spectrum) appeared at 5.6, 6.0 (vinyl carbon-linked hydrogen), and 1.8 ppm (methyl carbon-linked hydrogen at the vinyl groups) in all the SPVA spectra. This clearly indicates the presence of methacryloyl group as reported in the literature [S1]. This result implies that the SPVA is produced via trans-esterification between OH groups of PVA and GMA in aprotic DMSO solvent. As seen in the substitution degree and yield calculated from ^{1}H-NMR analysis (Table S1), the substitution yield was increased to 95% with increasing time up to 6 h and then leveled off. Thus, the reaction time of 6 h was selected as the optimum for trans-esterification in this system. Likewise, as shown in the ^{1}H-NMR spectra for the SPVA prepared with different GMA:PVA molar ratios (Fig. S2 (c) and Table S1), the trans-esterification reaction with different GMA:PVA molar ratios all exhibited substitution yields of more than 97%, indicating the occurrence of trans-esterification almost without unreacted GMA [S2]. Therefore, the SPVA with substitution degree ranging from 10 to 20 mol% can be precisely produced by the given trans-

esterification proceeding in aprotic DMSO at 60 °C for 6 h. Moreover, as presented in the FT-IR spectra for the further investigation on the functionality of SPVA with different GMA:PVA molar ratios (Fig. S2-c), the PVA spectrum exhibited peaks at 3340 cm^{-1} (-OH), 2935 cm^{-1} (CH), 1486 cm^{-1} (CH) and 1090 cm^{-1} (C-O) [S3], which were the characteristic functional groups of the PVA. On the other hand, for the SPVAs with different substitution degree, three peaks, indicative of the presence of methacryloyl groups, appeared at 1721 cm^{-1} (C=O), 1634 cm^{-1} (C=C), 1180 cm^{-1} (C-O) in all the spectra. Their intensities seemed to be slightly stronger, with increasing degree of substitution, while the OH intensity decreased [S4]. This result strongly supports the successful synthesis of SPVA via trans-esterification under the given conditions.

To investigate the radiation sensitivity of SPVA-18, the gel fraction and swelling degree of SPVA were measured to investigate its degree of crosslinking and stability in water, and these results are shown in Fig. S3. The gel fraction of the SPVA films was quantified by measuring the weight of insoluble parts after solvent extraction in DMSO for 3 d at 80 °C. The gel fraction (W_{gel}) was calculated using the equation $W_{gel}\,(\%) = W_1/W_0 \times 100$, where W_0 is the weight of the dried film before extraction, and W_1 is the dried weight of the film after extraction. The swelling degree of the irradiated SPVA films at (50, 100, and 200) kGy was quantified by measuring the weight of the swollen film after immersion in deionized water for 7 d at 50 °C and carefully wiping away the excess water with soft tissue paper. The swelling degree (SW) was calculated using the equation $SW\,(\%) = (W_s - W_d)/W_d \times 100$, where W_s is the weight of swollen SPVA film, and W_d is the weight of the dry SPVA film before immersion. As shown in Fig. S3, the irradiated SPVA-18 films (unlike the non-irradiated one) exhibited the gel fraction of 99% even at the absorbed dose of 50 kGy, indicating the efficient formation of a crosslinked network structure. Moreover, non-irradiated SPVA-18 film showed the swelling degree of 167%. In contrast, the swelling of

irradiated SPVA-18 gradually decreased to 0% with increasing absorbed does. This reduced swelling degree could be ascribed due to the fact that the formation of the crosslinking network structure significantly suppresses the water-driven swelling of SPVA-18. Its crosslinking density (depending on the absorbed dose) further maximizes the swelling suppression of irradiated SPVA, leading to the dose-dependent swelling degree of the irradiated SPVA-18 film [S5]. Therefore, SPVA-18 was selected as the new radiation-crosslinkable hydrophilic binder for the preparation of the crosslinked hydrophilic carbon electrodes in this study.

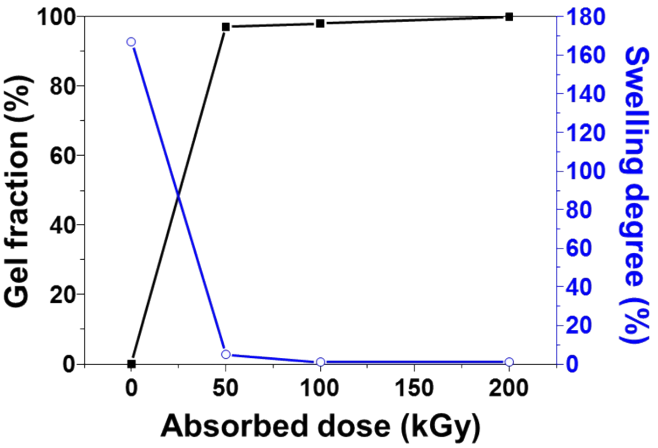

Fig. S3. Gel fraction and swelling degree of SPVA-18 film with different absorbed doses.

2. Solvent-extraction resistance of carbon electrodes

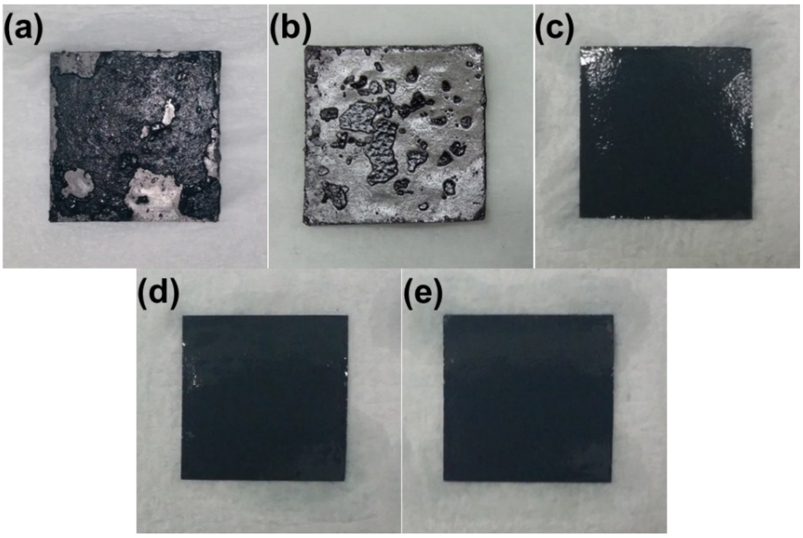

Fig. S4. Photographs of the experimental electrodes after solvent extraction: 10-PVDF-0 (a), 10-SPVA-0 (b), 10-SPVA-50 (c), 10-SPVA-100 (d), and 10-SPVA-200 (e).

3. Supporting references

[S1] E. G. Crispim, J. F. Piai, A. F. Rubira and E. C. Muniz, *Polym. Test*, 2006, **25**, 377-383.

[S2] A. V. Reis, A. R. Fajardo, I. T. A. Schuquel, M. R. Guilherme, G. J. Vidotti, A. F. Rubira and E. C. Muniz, *J. Org. Chem.*, 2009, **74**, 3750-3757.

[S3] H. S. Mansur, C. M. Sadahira, A. N. Souza and A. A. P. Mansur, *Mater. Sci. Eng. C*, 2008, **28**, 539-548.

[S4] L. Ferreira, M. M. Vidal, C. F. G. C. Geraldes and M. H. Gil, *Carbohyd. Polym.*, 2000, **41**, 15-24.

[S5] E. Jabbari and S. Nozari, *Eur. Polym. J.*, 2000, **36**, 2685-2692.
